# Effect of autogenous growth factors released from platelet concentrates on the osteogenic differentiation of periodontal ligament fibroblasts: a comparative study

**DOI:** 10.7717/peerj.7984

**Published:** 2019-10-31

**Authors:** Zheng Zhang, Xinyue Li, Jing Zhao, Wenjun Jia, Zuomin Wang

**Affiliations:** 1 Department of Periodontology, Tianjin Stomatological Hospital, Hospital of Stomatology, Nankai University, Tianjin, China; 2 Department of Stomatology, Beijing Chao-Yang Hospital, Capital Medical University, Beijing, China

**Keywords:** Platelet concentrates, Periodontal ligament fibroblasts, Osteogenic differentiation

## Abstract

**Background:**

Platelet concentrates have been used in tissue regeneration. The purpose of this study was to examine effects of growth factors released from leukocyte- and platelet-rich fibrin (L-PRF) and concentrated growth factor (CGF) on the osteogenic differentiation of periodontal ligament fibroblasts (PDLFs).

**Methods:**

Leukocyte- and platelet-rich fibrins, CGFs and PDLFs were obtained from New Zealand rabbits. The release of basic fibroblast growth factor (bFGF), bone morphogenetic protein 2 (BMP-2) and transforming growth factor β1 (TGF-β1) from L-PRFs and CGFs was measured at 5 h and 1, 3, 5, 7 days, using the enzyme linked immunosorbent assay. The PDLFs were treated with exudates of L-PRF or CGF. After the treatment, cell counting kit-8 assay was performed at day 1, 3, 5 and 7. Alkaline phosphatase (ALP) assay and Western blotting were applied at day 7. Three blocking antibodies were used to neutralize the proteins of bFGF, BMP-2 and TGF-β1.

**Results:**

Leukocyte- and platelet-rich fibrin and CGF showed different growth factor release pattern, but similar accumulated concentration of these growth factors. PDLFs proliferation was significantly promoted by both L-PRF and CGF at day 1, 3 and 7, and CGF group was superior to L-PRF group at day 1 and 3. Both L-PRF and CGF significantly enhanced PDLFs ALP activity and protein expression of osteogenic markers. The osteopontin level was higher in CGF group than in L-PRF group, but no significant differences were found between two groups for ALP activity. Three blocking antibodies significantly downregulated both L-PRF and CGF induced osteogenic markers expression.

**Conclusion:**

Both CGF and L-PRF can promote the proliferation and osteogenic differentiation of PDLFs. The bFGF, BMP-2 and TGF-β1 are involved in both L-PRF and CGF induced osteogenic differentiation of PDLFs.

## Introduction

Periodontitis is a highly prevalent inflammatory disease that involves destruction of periodontal support tissue such as the periodontal ligament, alveolar bone and cementum, and ultimately leading to tooth loss (Kim et al., 2013). The purpose of periodontal treatment is not only to control inflammation, but also to promote the regeneration of the destructed periodontal tissue to form a new attachment. It has been found that periodontal ligament fibroblasts (PDLFs) are able to exhibit a number of the phenotypic characteristics of osteoblasts, including osteocalcin (OCN), osteopontin (OPN) and osteriox (OSX) (Duan et al., 2017). In addition to constantly reconstructing alveolar bone, PDLFs also produce new dental cement and principal fibers, and synthesize the extracellular matrix (Choe et al., 2012; Zhang et al., 2013). For these reasons, PDLFs are believed to be key players during periodontal tissue regeneration. In fact, it is difficult to achieve a satisfactory result in periodontal tissue regeneration by conventional periodontal treatment. One possible reason is that residual healthy PDLFs at the periodontal lesion are very few due to the inflammatory damage, and conventional treatment methods lack of active induction to PDLFs (Kim et al., 2013).

Growth factors are bioactive proteins which have a critical role in cell proliferation (Hu et al., 2018) and osteoblastic differentiation (Yu & Wang, 2014). Among them, fibroblast growth factor (FGF) has key roles in regulating osteogenesis, and bone and mineral homeostasis (Ornitz & Marie, 2015). Bone morphogenetic protein (BMP) and transforming growth factor (TGF) are also known to be associated with bone formation (Yu & Wang, 2014). The treatment with these growth factors has been found to be efficient for stimulating PDLFs (Hyun et al., 2017). However, the human body is a complex biological environment, and PDLFs osteogenic differentiation is not regulated by one growth factor alone. Therefore, it is essential to identify an autologous source and effective growth factor group in PDLFs osteogenic induction. Platelet is one of the major resources of autogenous growth factors (Qiao, An & Ouyang, 2017). It has been reported that several growth factors, such as FGF, BMP, TGF-β and others, are contained in platelets (Kim et al., 2014). However, it is still not clear whether these growth factors in platelet concentrates contribute to the modulation of osteogenic differentiation of PDLFs.

In recent years, numerous platelet concentrates, such as platelet-rich plasma (PRP), leukocyte- and platelet-rich fibrin (L-PRF) and concentrated growth factor (CGF) have been used in dentistry (Sahin, Gokmenoglu & Kara, 2018). The application of PRP has gained great debate on its biosecurity and stability because of the artificial thrombin and anticoagulant additive (Hong, Chen & Jiang, 2018). L-PRF is a second-generation platelet concentrated product which does not require the addition of biological agents, and has no toxicity or immunogenicity (Duan et al., 2018). In 2006, Sacco (2006) introduced CGF, which is prepared by repeatedly switching the centrifugation speed using a special centrifugal machine. The different centrifugation speed permits the isolation of fibrin matrix that is markedly larger, denser and richer in growth factors as compared to L-PRF (Qiao & An, 2017). In theory, CGFs appear to exhibit superior potential for tissue regeneration. However, comparative studies between them have been conducted on osteogenic differentiation of stem cells of the apical papilla (Hong, Chen & Jiang, 2018) and animal bone defect repair (Park et al., 2016; Kim et al., 2014), only a few studies support this. Both L-PRF (Li et al., 2018; Duan et al., 2018) and CGF (Yu & Wang, 2014; Qiao & An, 2017; Li et al., 2019) have been reported to be effective in osteogenic differentiation of PDLFs. However, it has not been previously evaluated whether CGF used for PDLFs differentiation has better attributes than L-PRF.

Therefore, the main purpose of this study was to determine whether CGF is more effective in PDLFs differentiation than L-PRF. The secondary objective of this study was to evaluate whether growth factors in platelet concentrates, such as basic FGF (bFGF), BMP-2 and TGF-β1, can effectively function to induce osteogenic differentiation of PDLFs. Many animals, including dogs (Yu & Wang, 2014; Park et al., 2016), pigs (Jungbluth et al., 2014), rabbits (Kim et al., 2014) and rats (Qin et al., 2016; Duan et al., 2018), have been used to investigate the biological characteristics of platelet concentrates. Compared with larger animals (dogs and pigs), rabbits are relatively inexpensive to purchase, house and can be maintained in the setting of laboratories. Furthermore, rabbits are phylogenetically closer to humans than rodents (rats). Because of the limitations of larger animals and rodents, rabbits represent a better option in the present study.

## Materials and Methods

### L-PRF/CGF preparation

A total of four 4-month-old New Zealand rabbits were purchased from Xing Long Experimental Animal Center, Beijing, China. The experimental protocol was approved by the Ethical Committee for the Experimental Use of Animals of Tianjin Stomatological Hospital, Tianjin, China (2015-011). We collected 20 ml venous blood from the ear veins of each rabbits, and put the blood in four vacuum tubes containing no anticoagulant. Two tubes (Vacuette 454092; Greiner Bio-One, Kremsmunster, Austria) were used to produce L-PRFs by centrifuging at 3,000 rpm for 12 min (Eppendorf, Hamburg, Germany). And the other two (KJ040A; Kangjian Medical, Jiangsu, China) were centrifuged (Medifuge; Silfradentsrl, Sofia, Italy) to fabricate CGFs using a program with the following characteristics: 30-s acceleration, 2 min at 2,700 rpm, 4 min at 2,400 rpm, 4 min at 2,700 rpm, 3 min at 3,000 rpm and 36-s deceleration and stop.

### Cell isolation and cell culture

The rabbit PDLFs were obtained from extracted teeth. After rabbits were euthanized by intravenous injection of an overdose of sodium pentobarbital, the teeth were immediately extracted and rinsed with phosphate buffered saline. The periodontal ligament tissues attached to the middle one-third of the roots were removed by a surgical scalpel and then minced. Type I collagenase (one mg/ml; Gibco, Gaithersburg, MD, USA) was added and digestion was performed on a thermostat shaker (37 °C, 30 min). And then those digested tissue chunks were cultured in Dulbecco modified Eagle medium (DMEM; Gibco, Gaithersburg, MD, USA) with 10% fetal bovine serum (FBS; Hyclone, Logan, UT, USA), 100 U/ml penicillin (Genview, Houston, TX, USA) and 100 μg/ml streptomycin (Genview, Houston, TX, USA). The culture medium was replaced every 3 days after passaging, and cells of the third passage were used for the experiment. Cellular morphologies of PDLFs were observed by inverted phase contrast microscopy and then PDLFs were identified by vimentin (Abcam, Cambridge, MA, USA) and Pan Cytokeratin (PCK; Abcam, Cambridge, MA, USA) immunostaining.

### Growth factor measurement

Enzyme linked immunosorbent assay (ELISA) was utilized to quantify the concentration of growth factors released from L-PRF (*n* = 8) and CGF (*n* = 8) at 5 h and 1, 3, 5, 7 days. All L-PRF and CGF samples were placed in separate wells of a 12-well plate with two ml of cell culture media. The samples were incubated in a humidified incubator at 37 °C where growth factors were gradually released over time. At each time point, the supernatants from each well were collected and replaced with two ml fresh media. The supernatant concentrations of bFGF, BMP-2 and TGF-β1 released from the L-PRF and CGF were determined by ELISA kits (Huamei, Wuhan, China) following the manufacturer’s instructions. Absorbance was measured at 450 nm on a microplate reader.

### Treatment of PDLFs

The rabbit PDLFs were randomly divided into three groups: (1) control group (*n* = 8): PDLFs cultured with normal medium; (2) L-PRF group (*n* = 8): PDLFs cultured with L-PRF exudates; and (3) CGF group (*n* = 8): PDLFs cultured with CGF exudates. The method for the preparation of L-PRF/CGF exudates is as follows: the L-PRF and CGF membranous films were soaked in five ml fresh DMEM without FBS and incubated at 37 °C for 7 days. After incubation, the exudates were collected via centrifugation. DMEM enriched with exudates is the solution defined as the 100% exudates. Experiments were performed with 50% exudates.

### Cell counting kit-8 assay

The PDLFs were aliquoted into 96-well plates at a density of 1 × 10^3^ cells/well, and incubated overnight to allow cell attachment. After 1, 3, 5 and 7 days of incubation following the addition of L-PRF or CGF exudates, the cells in each well were incubated with 10 μl of cell counting kit-8 (Zomanbio, Beijing, China) for 1 h. The optical density values were measured using a Microplate Reader at 450 nm.

### Alkaline phosphatase activity

The PDLFs were seeded in two 12-well plates at a density of 1 × 10^5^ cells/well, and exposed to L-PRF or CGF exudates for 7 days, cells cultured in normal medium alone served as control group. Alkaline phosphatase (ALP) activity was determined by using ALP assay kit (Beyotime, Nanjing, China) according to the manufacturer’s instructions.

### Western blotting of OCN, OPN and OSX

The PDLFs were seeded into culture bottle at a density of 2 × 10^6^ cells/ bottle and cultured with the exudates of L-PRF and CGF for 7 days. Following centrifugation, PDLFs were lysed in radio-immunoprecipitation assay buffer, and proteins were separated by sodium dodecyl sulfate polyacrylamide gel electrophoresis and transferred to nitrocellulose membranes. The membranes were blocked with Blotto blocking solution at room temperature for 2 h and subsequently incubated overnight at 4 °C with primary antibodies (1:1,000; Abcam, Cambridge, MA, USA) against OPN, OCN and OSX. Then the membrane was incubated with the corresponding secondary antibody for 1.5 h, and the immunoreactive band was detected with the Western Lightning^®^-ECL, Enhanced Chemiluminescence Substrate (NEL100001EA; Perkin Elmer, Waltham, MA, USA). The optical densities of the bands were quantified using Labworks TM Analysis Software (UVP, Upland, CA, USA).

### Blocking assays of bFGF, BMP-2 and TGF-β1

To determine whether bFGF, BMP-2 and/or TGF-β1 involved in L-PRF and CGF induced osteogenic differentiation markers (OCN, OPN and OSX) expression, we used three blocking antibodies (Affinity Biosciences, Cincinnati, OH, USA) to neutralize the proteins of bFGF, BMP-2 and TGF-β1. Briefly, prior to the addition of L-PRF and CGF exudates, the PDLFs were incubated for 1 h with fresh media containing 20 μg/ml of anti-bFGF, anti-BMP-2 or anti-TGF-β1 antibody. An equal amount of isotype control IgG was used as the negative control. Afterward, the PDLFs were exposed to L-PRF or CGF exudates. After 7 days of culture, the cells were harvested for western blotting assay.

### Statistical analysis

Statistical analyses were performed using SPSS 13.0 software (SPSS Inc., Chicago, IL, USA). All data are presented as the mean ± standard deviation. A two-tailed Student’s *t*-test or one-way ANOVA with Bonferroni correction was used for normally distributed data. Mann–Whitney U test or Kruskal–Wallis test was used for data that were not normally distributed. *P*-values less than 0.05 were considered statistically significant.

## Results

### Identification of PDLFs

**Figure 1:**
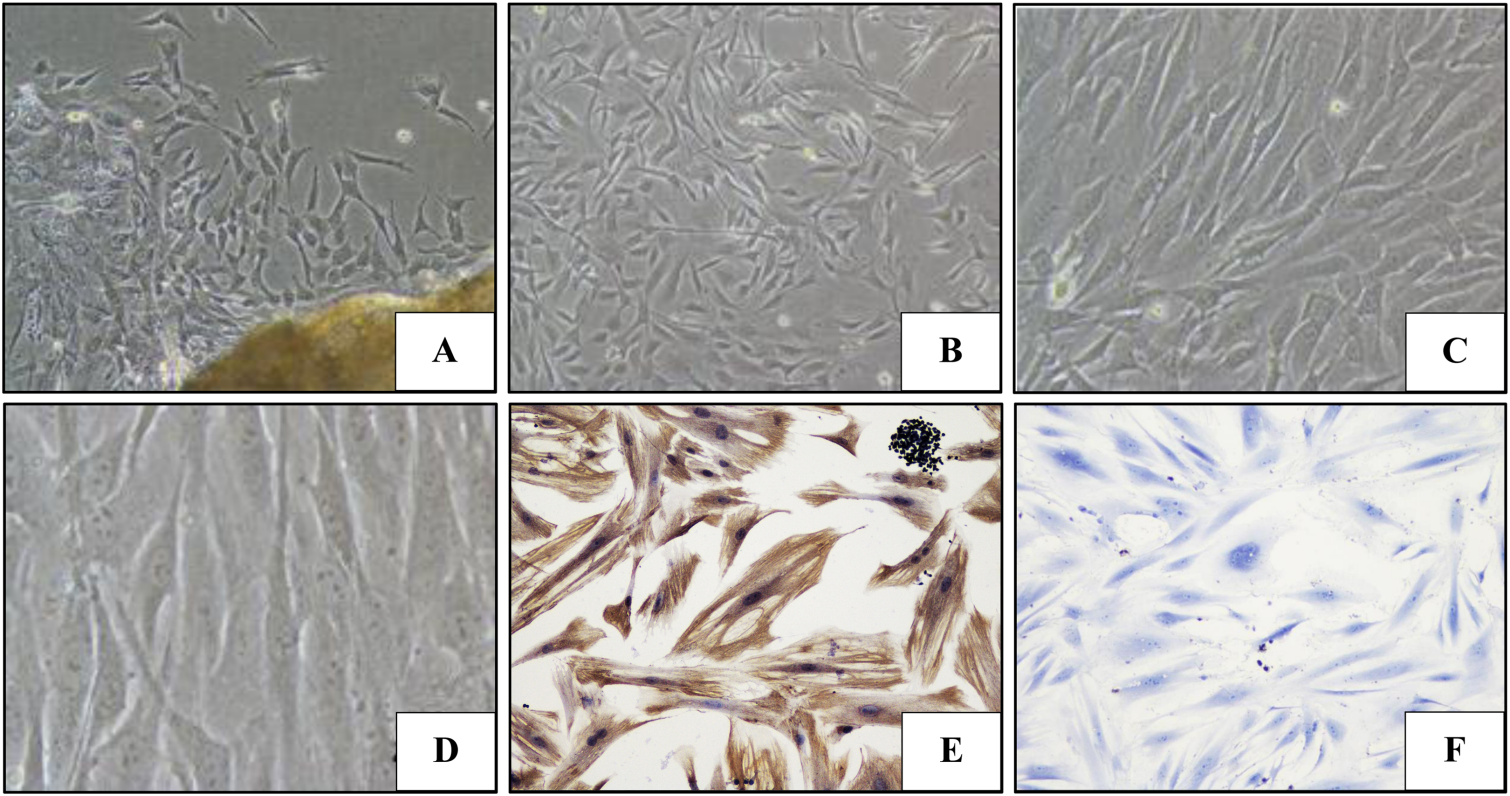
The morphology and immunohistochemical staining of PDLFs. (A–D) Cell morphous was observed with an inverted microscope (A and B: 10×; C: 20×; D: 40×). PDLFs showed a uniform, long spindle shape, with round or egg-shaped nuclei; (E) Vimentin was positive in the cytoplasm of PDLFs (20×); (F) Pan Cytokeratin (PCK) was not expressed in PDLFs (20×).

Periodontal ligament fibroblasts began to migrate from the edge of the tissues after 4 days of culture, and arranged radially around a central tissue block (Fig. 1A). The cultured cells reached approximately 70% confluence at 8 days, and reached almost complete confluence at about 10 days. The morphology of cells became uniform, with a long spindle shape (Figs. 1B and 1C) and round or egg-shaped nuclei (Fig. 1D). Immunohistochemistry testing of the PDLFs revealed that the cytoplasm was positive for vimentin (Fig. 1E); however, PCK was not expressed in the cytoplasm (Fig. 1F).

### Growth factor concentrations in the L-PRF and CGF supernatants

**Figure 2:**
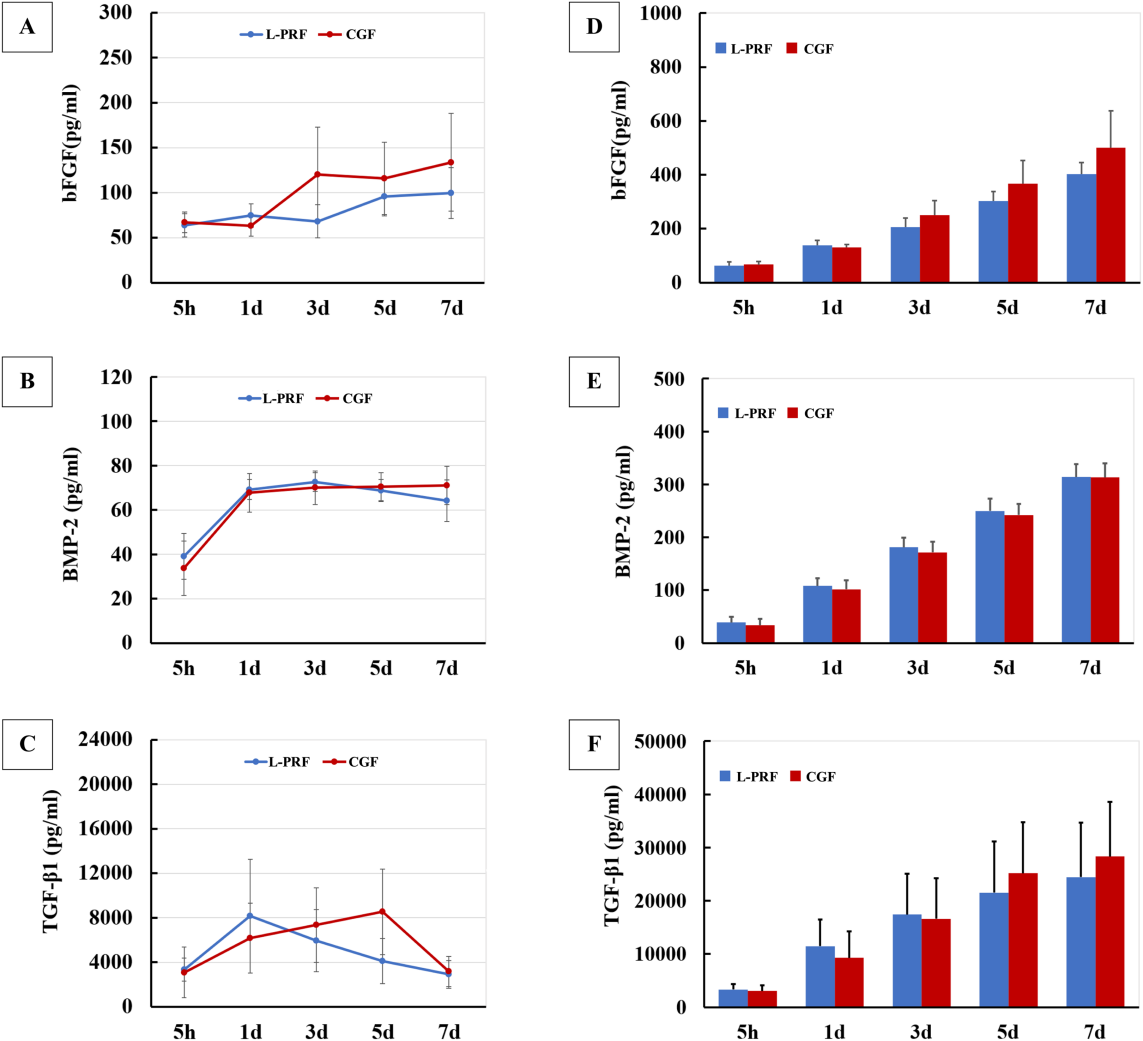
Growth factor concentration (*n* = 8 for each group and time point). (A–C) Growth factor concentrations in culture medium released from L-PRF and CGF at different time points; (D–F) The accumulated growth factor concentrations, which represent the sum of concentrations at each time point before next detection, were calculated.

The bFGF concentration remained stable in two groups at the early study time, followed by a dramatic increase in CGF group at day 3, and in L-PRF group at day 5 (Fig. 2A). For BMP-2 concentration, the general trend of the two evaluated groups at each time point was similar. The concentration increased dramatically at day 1 and remained stable (between 60 and 80 pg/ml) at later time points (Fig. 2B). The TGF-β1 concentration of L-PRF showed a dramatic increase at day 1, followed by a gradual decline for the remaining time points. Whereas CGF exhibited a gradual increase in TGF-β1 concentration from 5 h to day 5, followed by a rapid decline at day 7 (Fig. 2C). Additionally, we also calculated the accumulated growth factor concentrations which represent the sum of concentrations at each time point before next detection. As shown in Figs. 2D–2F, the accumulated concentrations of bFGF, BMP-2 and TGF-β1 increased continuously over the study time. No significant differences were found between the L-PRF and CGF groups at each time point for the accumulated concentration of all three growth factors.

### Effects of L-PRF and CGF on PDLFs proliferation

**Figure 3:**
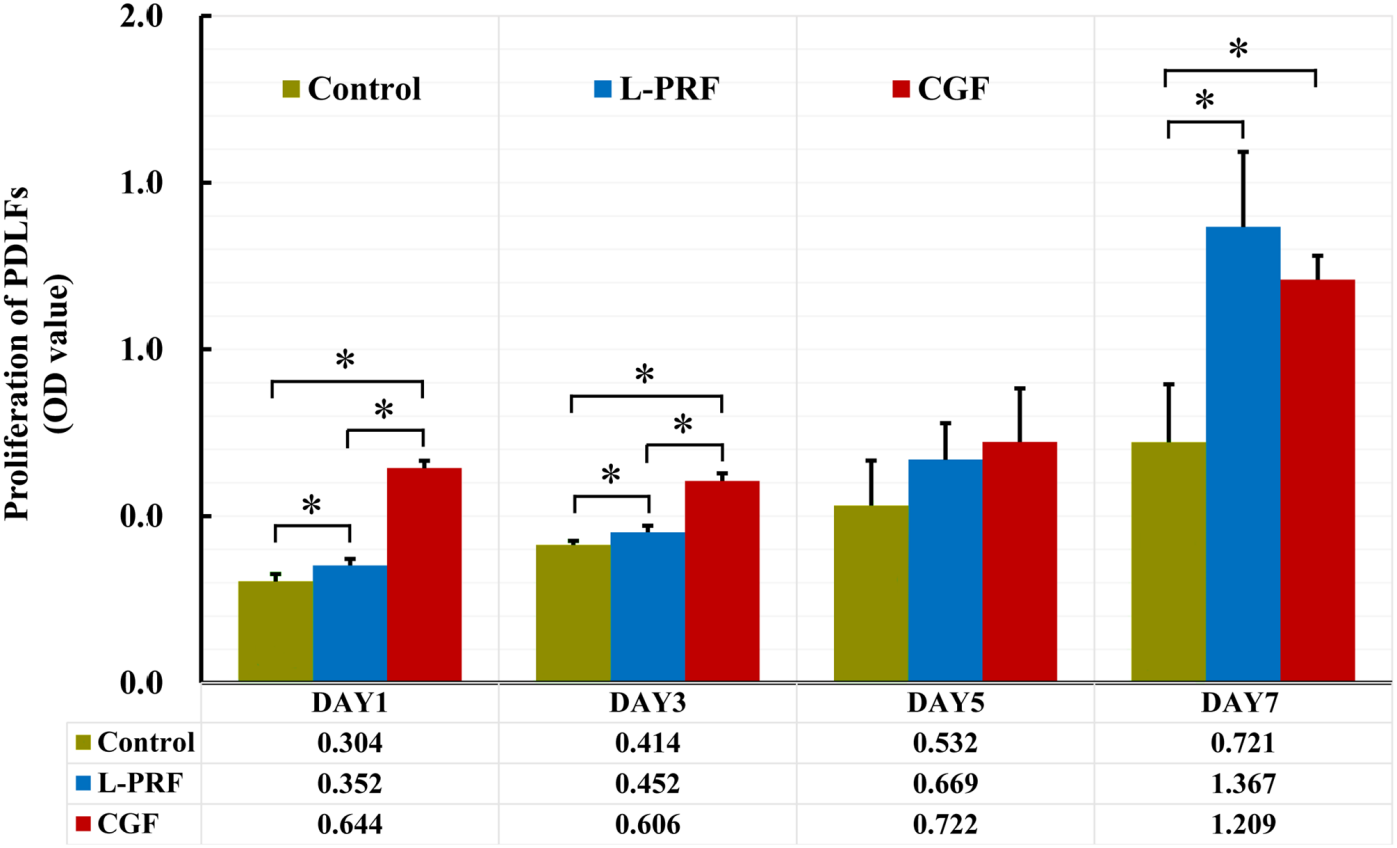
Effects of L-PRF and CGF on PDLFs proliferation at different time points (*n* = 8 for each group and time point). Proliferation of cells were measured using CCK-8 assay. PDLFs proliferation was significantly promoted by both L-PRF and CGF at day 1, 3 and 7, and CGF group was superior to L-PRF group at day 1 and 3; *P < 0.05.

As shown in Fig. 3, there was a statistically significant increase in PDLFs proliferation when the cells cultured with L-PRF or CGF exudates compared to control cells at three time points (day 1, 3 and 7). In addition, the proliferation of the PDLFs in CGF group is significantly higher than the cells in L-PRF group at day 1 and 3, whereas the difference was disappeared at later time points (day 5 and 7).

### Effects of L-PRF and CGF on ALP activity of PDLFs

**Figure 4:**
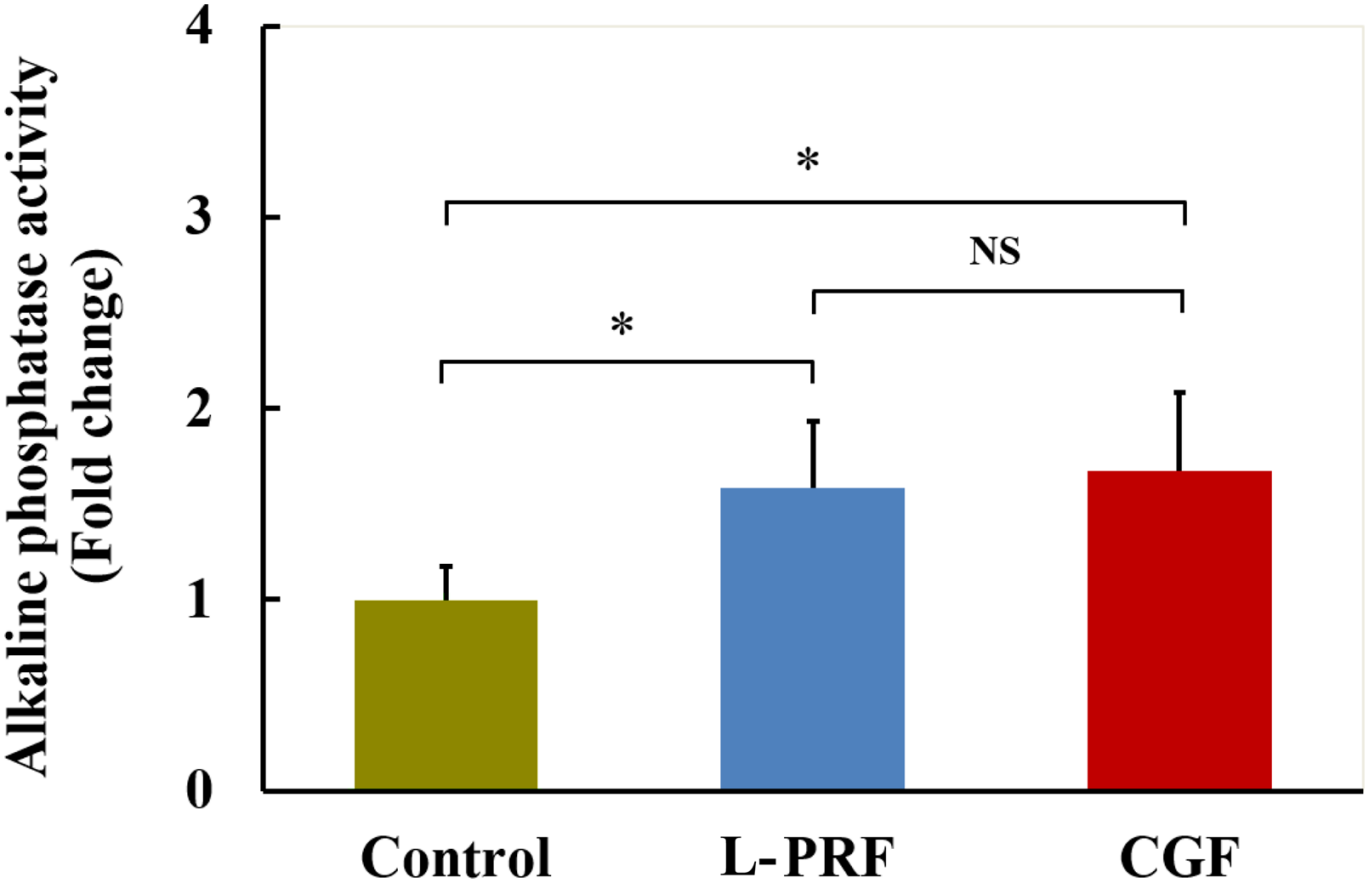
Effects of L-PRF and CGF on ALP activity of PDLFs (*n* = 8 for each group). After treatment with exudates of L-PRF or CGF, the ALP activity of PDLFs was significantly higher than that of the control group, whereas there was no significant difference between the CGF and L-PRF groups; *P < 0.05.

The effects of L-PRF and CGF on ALP activity of PDLFs are shown in Fig. 4. After treatment with exudates of CGF or L-PRF, the ALP activity of PDLFs was significantly higher than that of the control group (*P* < 0.05), whereas there was no significant difference between the CGF and L-PRF groups.

### Effects of L-PRF and CGF on OCN, OPN and OSX expression of PDLFs

**Figure 5:**
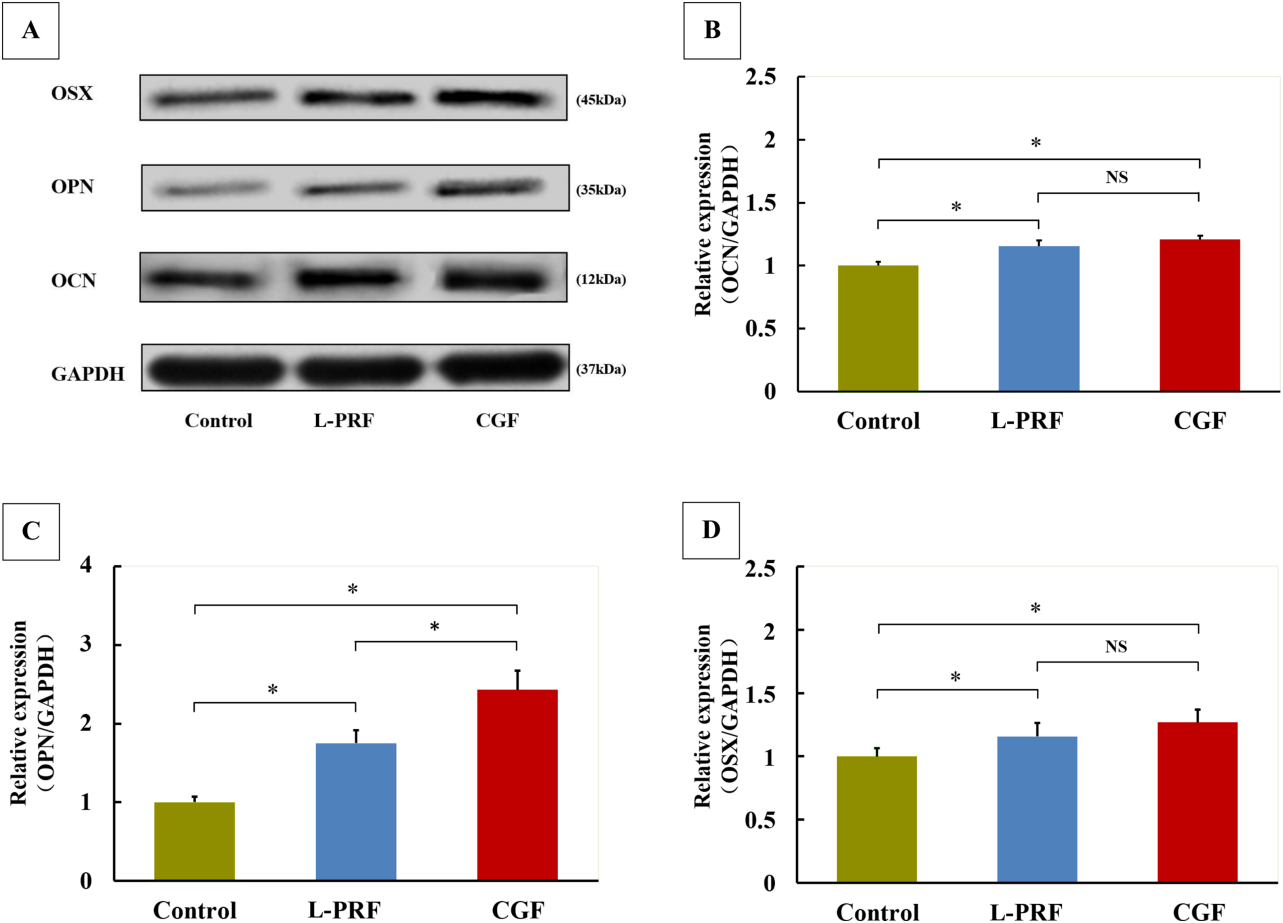
Effects of L-PRF and CGF on osteogenic markers expression of PDLFs (*n* = 4 for each group). Expressions of osteocalcin (OCN), osteopontin (OPN) and osteriox (OSX) were detected using western blot methods. The PDLFs treated with exudates of L-PRF or CGF significantly enhanced protein expression of OCN (A and B), OPN (A and C) and OSX (A and D), and the OPN level was significantly higher in CGF group than in L-PRF group (A and C); *P < 0.05.

The results from the western blotting experiments are shown in Fig. 5. After 7 days of treatment, OCN, OPN and OSX expression levels were 1.16, 1.75 and 1.16 times higher in L-PRF group, and 1.21, 2.23 and 1.27 times higher in CGF group compared to those in the control group. However, only the expression of OPN was significantly increased in CGF group compared to L-PRF group.

### Roles of growth factors in L-PRF and CGF induced OCN, OPN and OSX expression of PDLFs

**Figure 6:**
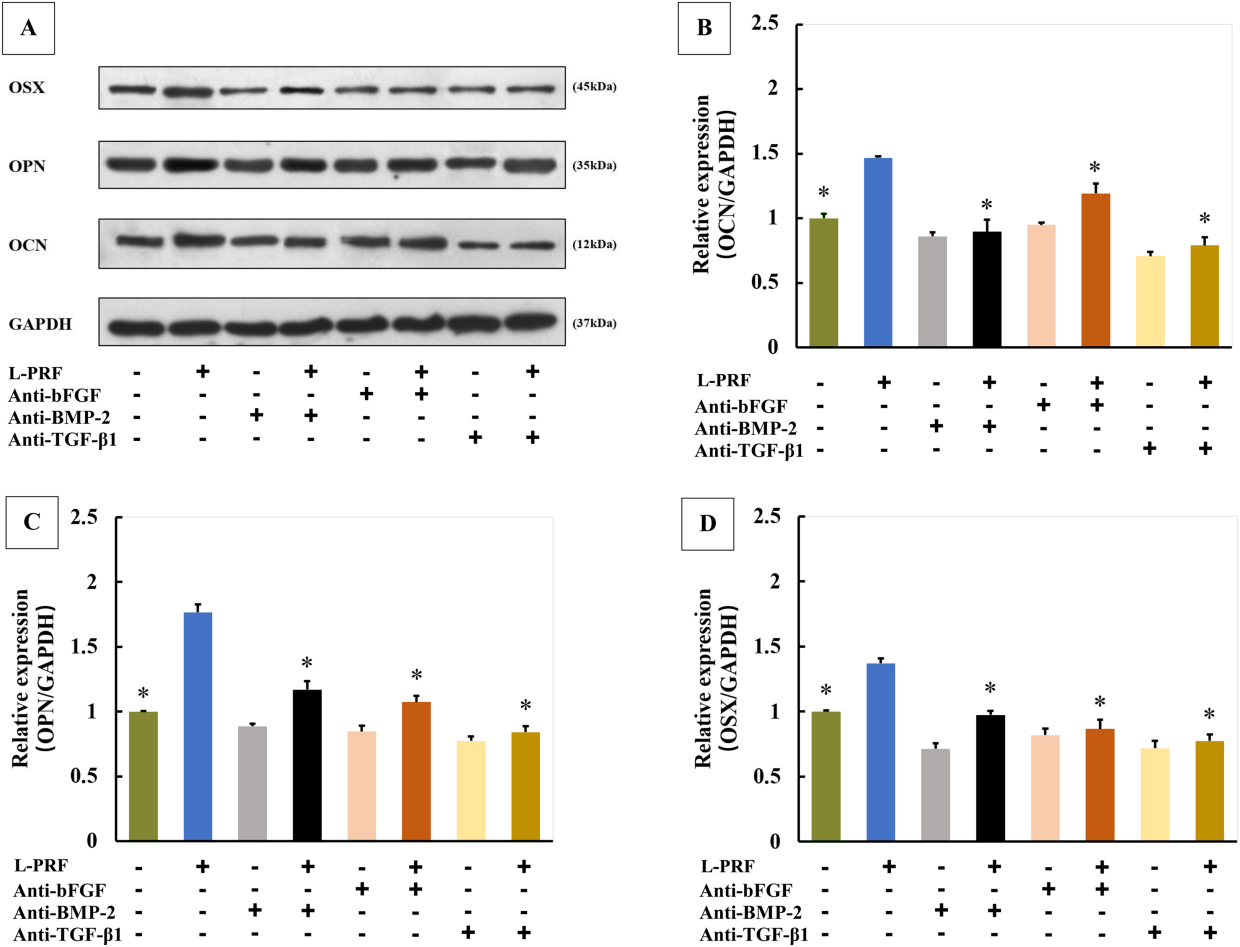
Roles of growth factors in L-PRF induced osteogenic markers expression of PDLFs (*n* = 4 for each group). Three blocking antibodies significantly downregulated L-PRF induced osteogenic markers expression (A–D); *P < 0.05 compared to L-PRF group.

**Figure 7:**
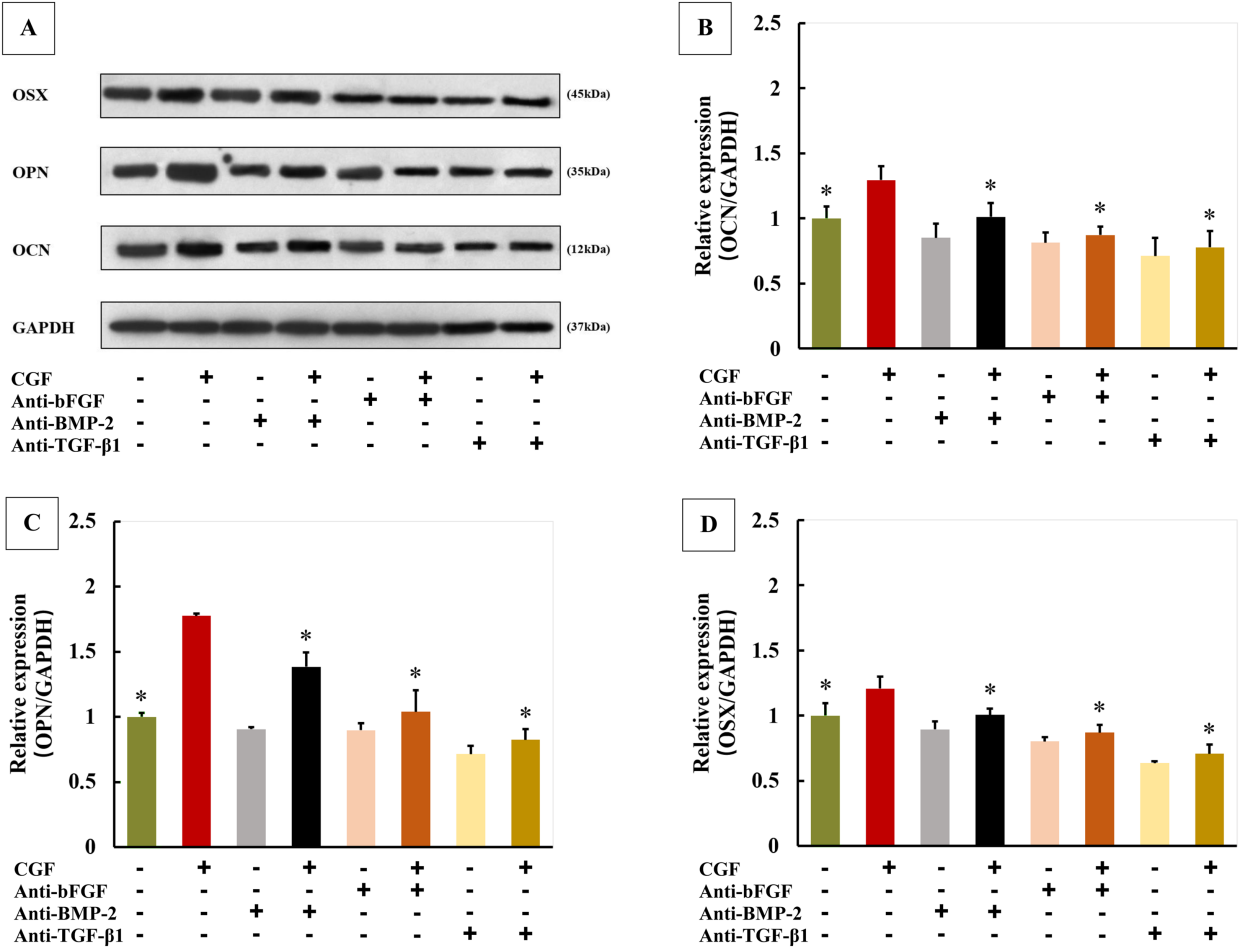
Roles of growth factors in CGF induced osteogenic markers expression of PDLFs (*n* = 4 for each group). Three blocking antibodies significantly downregulated CGF induced osteogenic markers expression (A–D); *P < 0.05 compared to CGF group.

The expressions of OCN, OPN and OSX were greatly increased in cells cultured with L-PRF exudates, and these inducible effects of L-PRF were significantly blocked by co-culture with anti-bFGF, anti-BMP-2 or anti-TGF-β1 antibody (Figs. 6A–6D). Similarly, CGF induced OCN, OPN and OSX expressions were also blocked by neutralization of bFGF, BMP-2 or TGF-β1 (Figs. 7A–7D).

## Discussion

In the present study, a slow release of BMP-2 was detected during at least 7 days in both L-PRF and CGF, even if the quantities remained quite small. It is interesting to notice that small quantities of BMP-2 has also been detected in L-PRF (Dohan Ehrenfest et al., 2018) and CGF (Bonazza et al., 2018) in previous publications. It has been reported that the different cell populations (mostly leukocytes) living in the fibrin clot may contribute to the release of BMP-2 (Dohan Ehrenfest et al., 2018). Release patterns of the growth factors analyzed in our study show some important differences, especially for TGF-β1. L-PRF reached the peak level of TGF-β1 at the time point of day 1. In contrast, CGF reached the peak level of TGF-β1 at the time point of day 5. CGF delayed the peak point to 4 days later than that of L-PRF. Similar studies with canine L-PRF provide comparable results, demonstrating that the majority of TGF-β1 is released from canine L-PRF in 24 h (Birdwhistell, Karumbaiah & Franklin, 2018; Visser et al., 2010). Additionally, a previous study in rats has demonstrated that the level of TGF-β1 released from CGF markedly increased and reached the highest amount at day 5, then decreased dramatically (Qin et al., 2016), which is consistent with our findings. The consistency in the results from multiple studies regarding the release of growth factor from L-PRF or CGF suggests that growth factor release pattern of CGF is different from that of L-PRF.

Concentrated growth factor has been thought to contain higher concentrations of certain growth factors due to its special centrifuge (Park et al., 2016). However, the accumulated concentrations of all three growth factors (bFGF, BMP-2 and TGF-β1) did not differ significantly between CGF and L-PRF in our study. The result is in accordance with the earlier human studies that demonstrated no significant difference in bFGF and TGF-β1 levels between CGF and L-PRF (Qiao, An & Ouyang, 2017; Masuki et al., 2016). These growth factors secreted by platelet concentrates play a crucial role in cell proliferation and differentiation, and contribute in some way to the positive clinical effects of platelet concentrates during bone regeneration (Dohan Ehrenfest et al., 2018). Therefore, our findings make it reasonable to hypothesize that the clinical effect of CGF may be similar to L-PRF on bone regeneration. This hypothesis is consistent with a previous observation demonstrating a similar beneficial effect of two platelet concentrates (L-PRF and CGF) on reconstruction of bony defects (Kim et al., 2014).

The proliferation of PDLFs is essential for periodontal tissue regeneration. In this study, L-PRF was found to promote the proliferation of PDLFs in vitro, and similar results were reported with other types of mesenchymal stem cells, including human dental pulp cells (Huang et al., 2010), human stem cells of the apical papilla (Hong, Chen & Jiang, 2018) and human oral bone mesenchymal stem cells (Dohan Ehrenfest et al., 2010). In addition, CGF has been reported previously to significantly increase the proliferation of human PDLFs (Qiao & An, 2017), beagle PDLFs (Yu & Wang, 2014) and rat Schwann cell (Qin et al., 2016). In the present study, we also found that CGF could remarkably stimulate the proliferation of rabbit PDLFs. Furthermore, the proliferation value in the CGF treated group was significantly higher than that of the L-PRF group at day 1 and 3, but this value in the CGF treated group was no longer higher at later time points (day 5 and 7). These results indicated that CGF and L-PRF may have a similar effect on the proliferation of PDLFs.

In this study, both CGF and L-PRF significantly enhanced PDLFs ALP activity and OCN, OPN and OSX protein levels. ALP is a unique substance expressed by osteoblasts during bone formation, whose concentration can often reflect the capacity of bone formation (Zhou, Liu & Xu, 2018). Thus, our findings suggested that both CGF and L-PRF could enhance PDLFs osteogenic differentiation. Similar results showed that L-PRF could promote the osteogenic differentiation of human dental pulp cells (Huang et al., 2010), osteoblasts (He et al., 2009) and human oral bone mesenchymal stem cells (Dohan Ehrenfest et al., 2010). Previous studies also demonstrated that CGF could stimulate osteogenic differentiation of beagle periodontal ligament stem cells (Yu & Wang, 2014) and rat bone marrow mesenchymal stem cells (Takeda et al., 2015). In addition, our study only showed a higher protein expression in CGF group for OPN, but not OCN and OSX, when compared with those in L-PRF group. Furthermore, no significant difference was also found between the L-PRF and CGF groups for ALP activity. Therefore, CGF was not found to be significantly superior to L-PRF, from the aspects of induction of osteogenic differentiation in our study.

The members of TGF-β super family such as TGF-β1 and BMPs have been reported to regulate osteoblastic differentiation in human PDLFs (Wei et al., 2017). In the present study, three growth factors have been found in both CGF and L-PRF, including bFGF, BMP-2 and TGF-β1. Thus, we hypothesized that CGF and L-PRF may upregulate OCN, OPN and OSX expression and promote osteogenic differentiation by regulation of growth factors released from them. To confirm this hypothesis, three blocking antibodies were used to abolish the stimulatory effects of CGF and L-PRF on osteogenic differentiation in PDLFs. The results showed that anti-bFGF, anti-BMP-2 or anti-TGF-β1 antibody significantly reduced OCN, OPN and OSX expression. These data demonstrated that all these three growth factors play critical roles in both L-PRF and CGF induced PDLFs osteogenic differentiation.

Several limitations of this study should be acknowledged. One limitation of our study is that we only evaluated the release of bFGF, BMP-2 and TGF-β1. However, there are numerous other growth factors associated with platelet concentrates that may function synergistically to benefit osteogenesis. Another shortcoming of this study is that we evaluated growth factor release and PDLFs biological characteristics for only 7 days. This timeline was based on a previous study (Hong, Chen & Jiang, 2018), but there was still notable growth factor release from platelet concentrates at day 8 as described in a previous study (Bonazza et al., 2015).

## Conclusions

The results from the present study demonstrated that CGF releases a similar amount of growth factor as L-PRF. Both CGF and L-PRF can promote the proliferation and osteogenic differentiation of PDLFs, and they seem to have no significant difference. Furthermore, the bFGF, BMP-2 and TGF-β1 are involved in both L-PRF and CGF induced osteogenic differentiation of PDLFs.

## Supplemental Information

10.7717/peerj.7984/supp-1 Supplemental Information 1 Raw data.

10.7717/peerj.7984/supp-2 Supplemental Information 2 Full-length uncropped blots (Figs. 5 and 6).

## Additional Information and Declarations

### Data Availability

The following information was supplied regarding data availability: The raw data are available in the Supplemental Files.
