# Supplementary figures and images for: Effect of autogenous growth factors released from platelet concentrates on the osteogenic differentiation of periodontal ligament fibroblasts: a comparative study
Source: PeerJ. 2019 Oct 31;7:e7984. doi: 10.7717/peerj.7984 (PMC6825745; doi:10.7717/peerj.7984)

## Slide 1
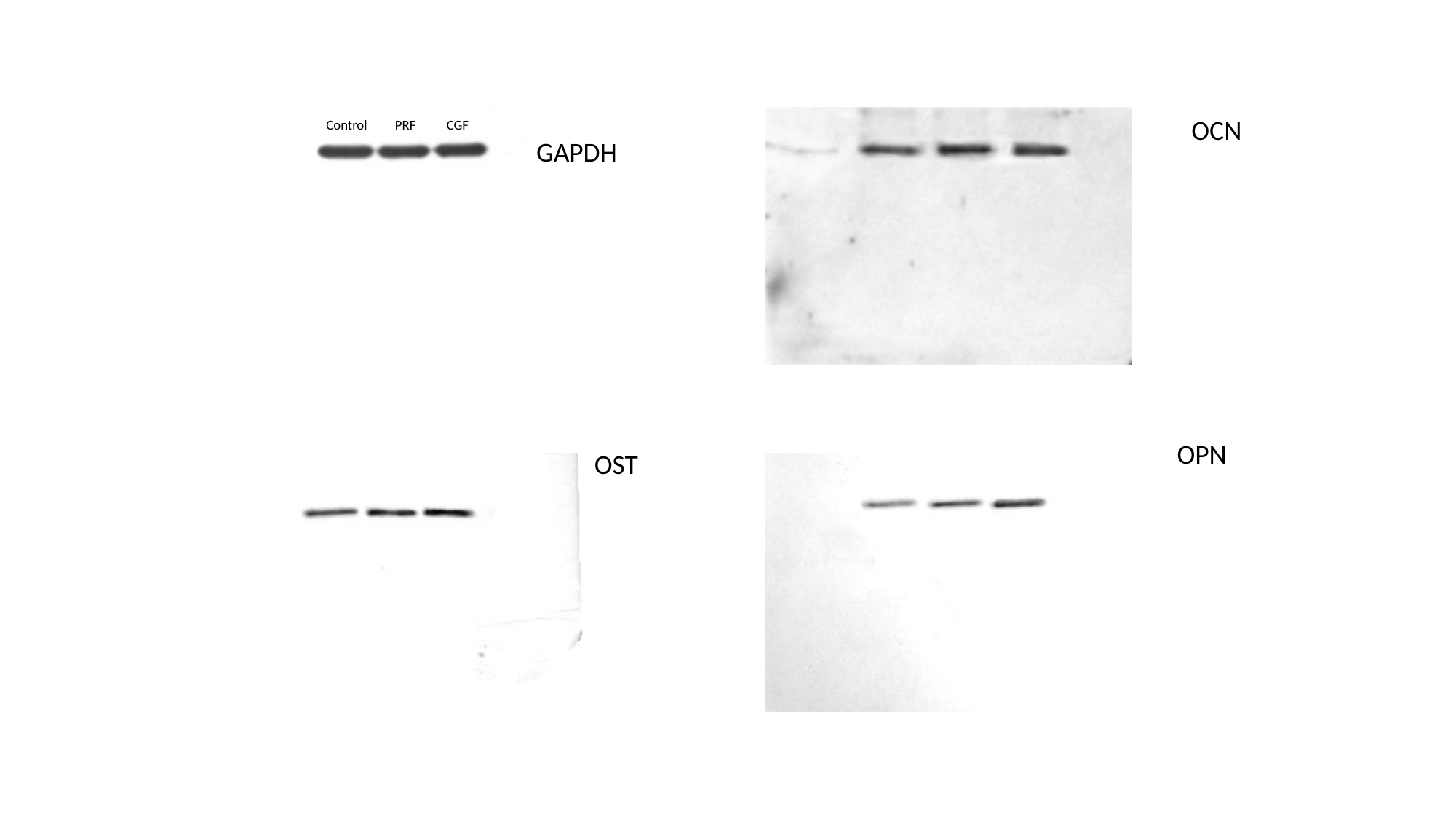

OCN
Control PRF CGF
GAPDH
OPN
OST

Supplement: Supplemental Information 2 [file peerj-07-7984-s002.zip › Full-length uncropped blots (Figs. 5 and 6)/Figure 5.pptx]

## Slide 1
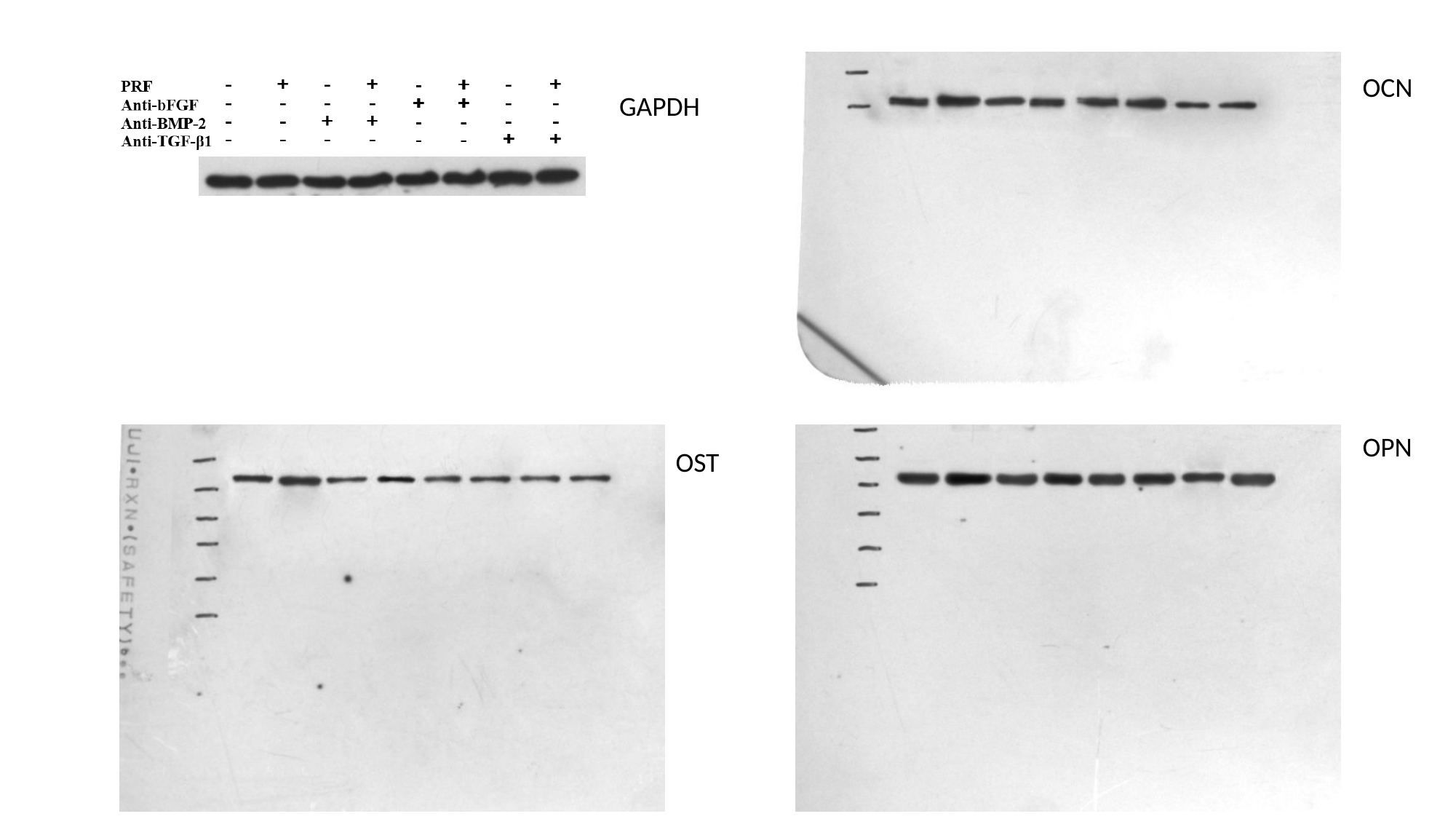

OCN
GAPDH
OPN
OST

## Slide 2
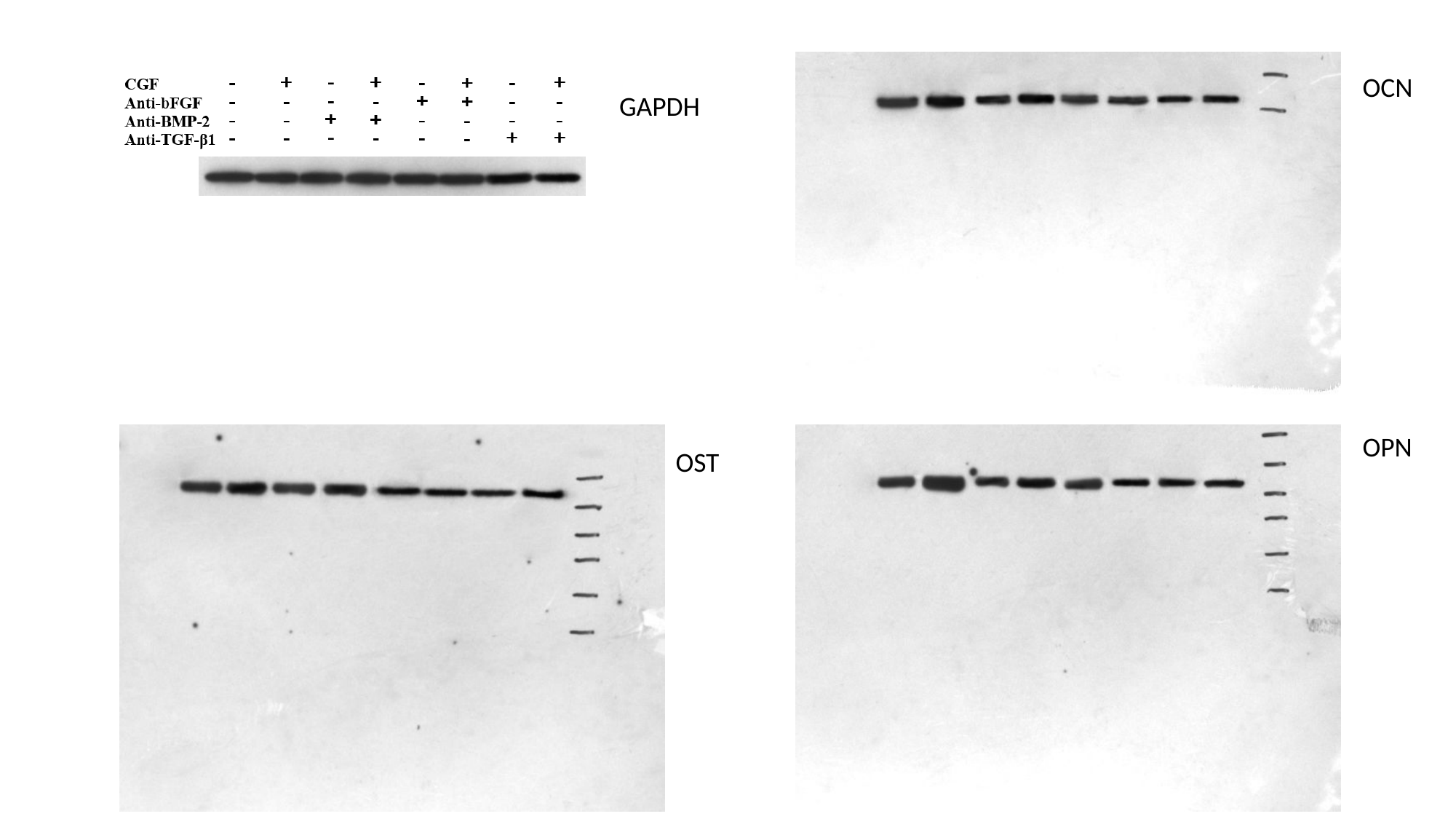

OCN
GAPDH
OPN
OST

Supplement: Supplemental Information 2 [file peerj-07-7984-s002.zip › Full-length uncropped blots (Figs. 5 and 6)/Figure 6.pptx]
